# Some Public-Health Questions Discussed

**Published:** 1921-07-30

**Authors:** 


### THE PUBLIC WELL-BEING.

### Some Public-Health Questions Discussed.

### INDUSTRIAL MEDICINE AND THE COMMUNITY.

At the British Medical Association meeting at Newcastle very interesting discussions have taken place on the important questions of public health, which are engaging the attention of medical men and social workers. There is no greater authority on the relation of medicine to industry than Mr. Edgar Leight Collis, who on July 21 opened a discussion on "The Importance of Industrial Medicine to the Community." Sir Thomas Oliver, introducing him, said that Professor Collis was the first whole-time professor of preventive medicine in this country. He hoped it was the inauguration of a new order of things in our Universities. He did not think a more appropriate choice could have been made than that of Professor Collis, whose long experience of factory work in the Home Office enables him to speak to the students of the University of Wales with an authority possessed by very few men. Professor Collis, in his paper, pointed out that fresh air, sunlight, cleanliness, and wholesome food are as necessary to the efficiency of adults as to the health of children. Careful inquiry shows that the underlying cause at the back of lost time is either certified sickness or lowered health preceding sickness. Industrial medicine properly applied could effect a saving each year on a conservative estimate of £140,000,000; by employing half the medical profession at £2,000 a year each for whole-time work a balance of £1,000,000 would be available for the State, though Mr. Collis hastened to assure his audience that such a wholesale engagement of the profession is not proposed or needed. Following the paper, Dr. W. H. Dickinson (Newcastle), one of the hon. secretaries of the Section, read a letter from Dr. T. M. Legge, of the Factory Department of the Home Office, deprecating the use of the term industrial medicine as suggesting a new branch of medicine, for which it will be necessary to obtain a special diploma. In his opinion it is merely the addition of some knowledge of the practical bearing of general medicine and surgery on the industrial conditions among which so large a proportion of the population spend their time. Every general practitioner is potentially an admirable industrial physician if he has the opportunity to exercise his skill behind the closed door of the factory and the workshop. How to open that door is the crux. Dr. Legge is convinced it should be opened widely only to those who have not lost touch with the practice of medicine, as it is their clinical knowledge which is wanted to help in detecting and treating the beginnings of disease. Industry already pays a biggish sum for health insurance, as to the benefits from which it may be a little dubious. Thus, from a factory employing 1,000 men every year considerably over £2,000 finds its way to the panel of doctors probably ignorant of the always interesting work their patients do. Dr. Legge urged that not until the door of the factory is open to medical men will there be scope for industrial medicine. Sir Kenneth Goadby said that in certain industries, for instance lead-working, doctors are hampered by the good-willed obstruction of employers and employed in efforts to improve their conditions. He dealt at length with lead-poisoning, and pointed out that lead operates on the progenitory system of men and women, and tends to a reduction in the birth-rate. The birth-rate for painters is 155, and the general figure is 162. He urged that medical men who are engaged in inquiry for the health of the nation should be met, not grudgingly, but whole-heartedly by Labour. Labour should not regard the statistics of the medical man as a sort of opportunity of prying into their private life. Captain Elliott, M.P., said that medical men should be treated by Labour as a medical man is treated in private—as a friend, and not as an employer's agent—who is only anxious to restore the man's health and send him back to work again. At the birth of a new branch of medicine it is important that the correct spirit should be attained. Dr. Harold Kerr (Medical Officer of Health, Newcastle) said the work in the canteens and public restaurants, and in the kitchens of hotels, is an industry in itself, and he drew attention to what is rather a paradoxical position. Under the Factory and Workshop Act a factory is distinguished from a workshop as being a place in which mechanical power is used. Between the stools of factory and workshop most of these kitchens fell, and the medical officer of health does not know quite whether an inspection of one of these places comes within his purview or not. The position should be defined, and places where food is prepared should come under the regular and effective supervision of the local public-health authority.

### LEGISLATION AND PUBLIC HEALTH.

Colonel Sir Thomas Oliver presided over the section dealing with "Preventive Medicine and Industrial Diseases." In introducing the speakers, he said Newcastle offers problems of interest to the medical officer of health and the student of industrial diseases. It has a welfare society, which is doing excellent work for the children, and, in addition, there is always the question of tuberculosis. The city has great difficulties as to overcrowding. On the question of the powers and responsibilities of local authorities, Dr. Modlin (Chairman of the Sunderland Health Committee) said he does not think one can wonder that local authorities, harassed by fear of ratepayers and increasing rates, and pressed by Government Orders, which are continually changing, hesitate to rush in without knowing what they are letting themselves in for. The health of the people, however, must be the first consideration. The Section, he urged, ought to consider whether the matter was best left to local authorities or to private efforts.

The Power of the Policeman.

A discussion, initiated by Capt. W. E. Elliott, M.C., M.P., took place on "The Effect of Health Legislation on the Health of the People." Discussing the history of health legislation, Capt. Elliott admitted that treatment has not advanced in the same degree as has legislation. Since 1850 the death-rate and the infantile-mortality rate have gone down, but he does not suggest that medical skill alone could have brought this about. It is due, he asserted, in no small way to the power of the policeman. The supply of water, first dirty water and then pure water, was one of the examples of the effect of legislation on the health of the people. The factory legislation has had an enormous effect also, and it was largely agitated for and driven home by laymen. Lord Shaftesbury was largely responsible, but not many public monuments are erected to him. There is not even a single portrait of him in the House of Commons. In many ways the compelling hand of the State is a great evil, but to deal with the manufacturers of the "forties" no less an authority than the State was capable of tackling the problem. The conditions under which the manufacturing population were allowed to live is still reflected in the North.

At present, Captain Elliott pointed out, there is a great wave of feeling against State interference of any kind. It would be fatal and tragic, however, if it allowed them to despise or ignore the great results which have come about from public-health legislation in the past. The lay population has forgotten the benefits, and resents the shepherding. That should be reduced to a minimum, but at the same time they should remember the great good that has been done by a very small amount of restrictive legislation. Diseases which have been regulated and sanitated and inspected have decreased, while others have not gone down proportionately. Measles, for instance, was responsible for 9,000 deaths in 1871, and the mortality is still 9,000 a year. As to the statistics for tuberculosis, Captain Elliott said he has tried to find sound reasons for the rapid post-war fall. It was said by some that the influenza epidemic, which swept the country like a broom, cleaned out all the weak chests; but the fact remained that non-pulmonary tuberculosis has declined also.

In the discussion, Dr. Modlin (Sunderland), Dr. Eustace Hill, Dr. Cope, Dr. Miller, Dr. McKail, and Dr. Dearden took part. Dr. Miller agreed it could not be denied that legislation has done much for public health; but the fault is too much red tape, too many forms to fill up. Legislation, he insisted, is wanted on useful lines, and the local authorities must be allowed to carry on the work with the least possible interference of the officials of the Government.

Colonel Sir Thomas Oliver (Newcastle), dealing with the relations of hospitals to municipal authorities, pointed out that they had a very good illustration in Newcastle, where there is a very large infirmary, in regard to which great anxiety for the future is felt. As a voluntary hospital, a few years ago, it was largely supported by what were called the better-class people in the district. Then the maintenance of the hospital was handed over, to some extent, to the working classes, who began to contribute so much per week. Personally, he had always felt that that was a risk, because labour troubles might come and the working-class contributions might not always continue. While they had to give the working classes greater representation owing to the money they are giving, those classes were getting, he might say, far more in return.

